# Phagocytosis of *Candida albicans* Inhibits Autophagic Flux in Macrophages

**DOI:** 10.1155/2018/4938649

**Published:** 2018-05-20

**Authors:** Zhimin Duan, Qing Chen, Leilei Du, Jianbo Tong, Song Xu, Rong Zeng, Yuting Ma, Xu Chen, Min Li

**Affiliations:** ^1^Institute of Dermatology, Jiangsu Key Laboratory of Molecular Biology for Skin Diseases and STIs, Chinese Academy of Medical Sciences and Peking Union Medical College, Nanjing 210042, China; ^2^Jiangsu Province Blood Center, Nanjing, Jiangsu 210042, China; ^3^Center for Systems Medicine, Institute of Basic Medical Sciences, Chinese Academy of Medical Sciences and Peking Union Medical College, Beijing 100005, China; ^4^Suzhou Institute of Systems Medicine, Suzhou, Jiangsu 215123, China

## Abstract

Autophagy machinery has roles in the defense against microorganisms such as *Candida albicans*. Lipidated LC3, the marker protein of autophagy, participates in the elimination of *C. albicans* by forming a single-membrane phagosome; this process is called LC3-associated phagocytosis (LAP). However, the influence of *C. albicans* on autophagic flux is not clear. In this study, we found that *C. albicans* inhibited LC3 turnover in macrophages. After the phagocytosis of *C. albicans* in macrophages, we observed fewer acridine orange-positive vacuoles and RFP-GFP-LC3 puncta without colocalization with phagocytized *C. albicans*. However, phagocytosis of *C. albicans* led to LC3 recruitment, but p62 and ATG9A did not colocalize with LC3 or *C. albicans*. These effects are due to an MTOR-independent pathway. Nevertheless, we found that the *C. albicans* pattern-associated molecular pattern *β*-glucan increased LC3 turnover. In addition, phagocytosis of *C. albicans* caused a decrease in BrdU incorporation. Blocking autophagic flux aggravated this effect. Our findings suggest that phagocytosis of *C. albicans* decreases autophagic flux but induces LAP in an MTOR-independent manner in macrophages. Occupation of LC3 by recruiting engulfed *C. albicans* might contribute to the inhibition of autophagic flux. Our study highlights the coordinated machinery between canonical autophagy and LAP that defends against *C. albicans* challenge.

## 1. Introduction

Although *C. albicans* is a normal constituent of the human microflora on the skin and in the oral cavity, intestines and vagina, an infection by this organism can increase morbidity and mortality. It is the fourth most common cause of nosocomial bloodstream infections [1, 2]. The use of immunosuppressant and indwelling medical devices, organ transplantation and HIV infection increase the probability of *C. albicans* infection and may cause a life-threatening disease [3]. Phagocytosis is a physiological cellular process that engulfs pathogens and degrades them in phagosomes [4]. As a major immune cell population to control *C. albicans* infection, macrophages can clear fungus by phagocytosis and produce proinflammatory cytokines upon recognizing pathogen-associated molecular pattern (PAMP) expressed on the surface of *C. albicans* wall by pattern recognition receptors (PRRs) [5].

Recently, researchers linked autophagy to the clearance of microorganisms, including viral, bacterial and fungal organisms [6, 7]. Autophagy is not only a process that maintains cellular homeostasis and metabolism, but also a key regulator of anti-*C. albicans* immunity [8–12]. For example, ATG7 or ATG5 mutants show increased sensitivity to *C. albicans* and increased mortality after systemic *C. albicans* infections [8, 13]. It is worth mentioning that microtubule-associated protein 1 light chain 3 (LC3), the marker protein of macroautophagy (referred as autophagy), participates in the elimination of *C. albicans* by forming a single-membrane phagosome. This process is called LC3-associated phagocytosis (LAP) [14], and its machinery is distinct from the canonical autophagy process. It was found that Dectin-1, a C-type lectin receptor, induces the recruitment of LC3 to phagosomes; Syk and reactive oxygen species (ROS) production were needed in *Saccharomyces cerevisiae*- and *β*-glucan particle-induced LAP, and *S. cerevisiae* could increase LAP in bone marrow-derived dendritic cells in a Syk-dependent manner [9, 15]. In addition, studies have found that the induction of autophagy reduces the phagocytosis of *S. cerevisiae* in murine macrophages [16]. However, the influence of *C. albicans* on the canonical autophagy process is not clear, and the connection between autophagy and LAP is undetermined.

In general, mechanistic target of rapamycin (MTOR) is a protein kinase that plays a crucial role in the regulating canonical autophagy [17]. MTOR participates in the formation of MTOR complex 1 (MTORC1) and MTOR complex 2 (MTORC2) [18, 19]. These two complexes utilize different substrates and evoke distinct downstream signalling to regulate cellular functions. The phosphorylation of MTORC1 activates the unc-51-like kinase 1 (ULK1) protein and negatively regulates autophagy. The function of MTORC2 is not fully understood, but it is thought to control MTORC1 signalling pathway and promote autophagy [20, 21]. Autophagy can take place in MTOR-independent manner. Classical autophagy regulators such as beclin-1 and the class III PI3K-associated protein Rubicon were found to be involved in *C. albicans*-induced LAP [22, 23]. However, the role of the crucial autophagy modulator MTOR and how it might regulate autophagy or LAP in macrophages in the context of *C. albicans* stimulation remain unclear.

To clarify the canonical autophagy regulation in macrophages upon phagocytosis of *C. albicans*, we determined LC3 turnover, RFP-GFP-LC3 puncta and acridine orange- (AO-) positive vacuole formation in THP-1-derived macrophages challenged with *C. albicans* spores. Furthermore, we detected the colocalization of LC3 and autophagy regulators such as p62, ATG9A, and Rubicon after phagocytosis of *C. albicans* in macrophages. In addition, we assayed whether MTOR signalling regulates autophagy in THP-1-derived macrophages that have phagocytized *C. albicans*. Finally, we explored the potential role of autophagy in the context of *C. albicans* stimulation.

## 2. Materials and Methods

### 2.1. *C. albicans* Strains

*C. albicans* (from China Medical Fungi Culture Collection Center) was cultured in SDA medium (2% glucose, 1% peptone, and 1.5% agar) overnight at 28°C to obtain yeast cells. The cells were washed twice with phosphate-buffered saline (PBS) and heat-killed for 30 minutes at 56°C. In all experiments (except when otherwise specified), dead microorganisms were used to avoid the differences in the changes in ratio between yeast and macrophage growth conditions. Calcofluor white (CFW) is a useful tool for analyzing the localization of *C. albicans* [24]. We used CFW, which exhibits fluorescence when exposed to ultraviolet light, to stain *C. albicans*.

### 2.2. Cells

Human monocyte cell line THP-1 (ATCC TIB-202, Manassas, VA, USA) was grown in RPMI-1640 growth medium (Gibco, Grand Island, NY, USA) supplemented with 10% fetal bovine serum (FBS) (Gibco), 100 IU/mL penicillin and 100 *μ*g/mL streptomycin solution in a humidified atmosphere of 5% CO_2_ at 37°C in an incubator. THP-1-derived macrophages are macrophage-like cells that are generated by treatment with phorbol-12-myristate-13-acetate (PMA) (Sigma-Aldrich, St. Louis, MO, USA) for 48 hours. Then, the cells were placed in a medium without PMA.

Murine macrophage-cell line RAW 264.7 (ATCC TIB-71, Manassas, VA, USA) was cultured in DMEM (Gibco) supplemented with 10% FBS, 100 IU/mL penicillin and 100 μg/mL streptomycin solution in a humidified incubator at 37°C and 5% CO_2_. The growth medium was refreshed every 3 days. When the cells reached 70%–80% confluence, they were treated with different stimuli.

### 2.3. Mice and BMDM Culture

C57BL/6 wild-type (WT) mice were maintained in specific pathogen-free facilities at the Institute of Systems Medicine (Suzhou, China). All animal studies were conducted under protocols approved by the Subcommittee on Animal Center at the Institute of Systems Medicine.

### 2.4. Bone Marrow-Derived Macrophage (BMDM) Isolation and Culture

Bone marrow cells were harvested from 6- to 9-week-old C57BL/6 mice by flushing the femurs and tibias with PBS and were then cultured in differentiation medium (DMEM containing 10% FBS, 50 ng/mL mouse M-CSF (Meltenyi, Bergisch Gladbach, Germany), 100 IU/mL penicillin and 100 *μ*g/mL streptomycin solution) for 7 days. The differentiation medium was changed every 3 days. BMDMs in 12-well tissue culture plates (5 × 10^5^ cells/well) were treated with live *C. albicans* spores (10 : 1, fungi to macrophages).

### 2.5. Reagents and Antibodies

The compounds used in this study included E-64d (E8640), pepstatin (P5318), rapamycin (V900930), chloroquine (C6628), dimethyl sulfoxide (D2650), and acridine orange (AO, A8097) (all from Sigma-Aldrich, St. Louis, MO, USA). Other compounds included pp242 (Abcam, Cambridge, MA, USA) and torin1 (Tocris, Bristol, UK). Primary antibodies included anti-LC3A/B (number 12741), anti-*β*-actin (number 8457), anti-MTOR (number 2983), anti-phospho-MTOR Ser2448 (number 5536), anti-phospho-MTOR Ser2481 (number 2974), anti-Rictor (number 2114), anti-phospho-Rictor Thr1135 (number 3806), anti-Raptor (number 2280), anti-phospho-Raptor Ser792 (number 2083), anti-4E-BP1 (number 9644), anti-phospho-4E-BP1 Thr37/46 (number 2855), anti-PKC*α* (number 2056), anti-phospho-PKC*α* Thr638 (number 9375), anti-phospho-ULK1 Ser555 (number 5869), anti-phospho-ULK1 Ser757 (number 6888), anti-ULK1 (number 4773), anti-phospho-p70 S6 kinase Thr389 (number 9234), anti-p70 S6 kinase (number 2708), anti-phospho-S6 ribosomal protein Ser240/244 (number 5364), anti-phospho-S6 ribosomal protein Ser235/236 (number 4858), anti-caspase-3 (number 9665), and anti-rabbit IgG (HRP-linked) (number 7074) (all from Cell Signaling Technology, Danvers, MA, USA). Antibodies against Rubicon (ab156052), ATG9A-Alexa Fluor 647 (ab206253), were purchased from Abcam. Alexa Fluor 647 goat anti-rabbit (A21245) and Alexa Fluor 568 goat anti-mouse (A11031) were purchased from Thermo Fisher Scientific (Waltham, MA, USA).

### 2.6. Western Blotting

Cells were lysed in RIPA Lysis Buffer containing protease inhibitor cocktail and the phosphatase inhibitor PhosSTOP (both from Roche Applied Science, Basel, Switzerland). Protein concentration was determined using the BCA assay kit (Beyotime Biotechnology, Haimen, Jiangsu, China). Equal amounts of protein from different samples were boiled and loaded onto 4–15% SDS-polyacrylamide gels (Bio-Rad Laboratories, Hercules, CA, USA). Proteins were transferred to PVDF membranes (Merck Millipore, Billerica, MA, USA), blocked with 5% bovine serum albumin (BSA) (AMRESCO, Solon, OH, USA) at room temperature, and stained with the indicated antibodies. The protein bands were visualized using chemiluminescence. The band intensities were quantified using Quantity One. *β*-Actin served as the loading control.

### 2.7. AO Staining Assay

The AO staining assay was used to label acidic vesicular organelles (AVO). The acidic compartment will emit as red while DNA will bind with AO and emit as green. The mean red/green fluorescence ratios were used to detect the autophagy level. After specific treatments, cells were washed with PBS for twice and incubated with AO (5 *μ*g/mL for 10minutes) at room temperature. Images were collected with an OLYMPUS FV1000 laser scanning confocal microscope (green: *λ*_ex_ = 488 nm and *λ*_em_ = 515 nm; red: *λ*_ex_ = 546 nm and *λ*_em_ = 620 nm). The red and green fluorescence intensities in the cells were determined by Quantity One.

### 2.8. Immunofluorescence Assays

The RFP-GFP-LC3B, GFP-LC3B and RFP-p62 transgenes were transfected into cells using the Premo Autophagy Sensor BacMam 2.0 system RFP-GFP-LC3B kit (P36239, Invitrogen Corp., Carlsbad, CA, USA), LC3B-GFP (P36235, Invitrogen Corp.) and p62-RFP (P36241, Invitrogen Corp.) according to the manufacturer's instructions. The RFP-GFP-LC3B makes it possible to distinguish autophagosomes and autolysosomes, because the acid-sensitive GFP fluorescence losses after autophagosomes fuse with lysosomes and the pH drops, while RFP is acid insensitive and maintains positive in both autophagosomes and autolysosome. LC3B-GFP is used to observe the general location for LC3B. Chloroquine (CQ) was used to evaluate the sensitivity of THP-1-derived macrophages to this system. Before detection, cells were transfected for at least 24 hours. GFP scanning, *λ*_ex_ = 488 nm and *λ*_em_ = 508 nm; RFP scanning, *λ*_ex_ = 555 nm and *λ*_em_ = 584 nm. LysoTracker probes (Thermo Fisher Scientific, L12492) were added to the culture medium at 50 nM, and the cells were incubated for 30 minutes at 37°C (*λ*_ex_ = 647 nm and *λ*_em_ = 668 nm). Cells were imaged using an OLYMPUS FV1000 laser scanning confocal microscope.

### 2.9. ROS Assay

ROS production was identified using a ROS Detection Kit (Beyotime Biotechnology). Cells were cultured in a 12-well plate at 37°C and 5% CO_2_. After treatment, 10 *μ*M DCFH-DA (diluted in serum-free culture medium) was added to the cells, which were incubated for 20 minutes at 37°C. Cells were harvested by trypsinization and washed twice with PBS. Samples were assayed on a flow cytometer using the 488 nm laser for excitation and detected at 525 nm. Rosup (50 *μ*g/mL) served as the positive control.

### 2.10. Annexin V-FITC and Propidium Iodide (PI) Staining by Flow Cytometry

Apoptosis of THP-1-derived macrophages was identified using an Annexin V-FITC Apoptosis Detection Kit (Beyotime Biotechnology) by flow cytometry. After treatment, the cells were washed with cold PBS. The cells were resuspended in binding buffer at a concentration of 1 × 10^6^ cells/mL. 5 *μ*L of Annexin V-FITC staining solution and 10 *μ*L of PI staining solution were added and then gently swirled to mix and incubated for 20 minutes at room temperature in the dark. 400 *μ*L of binding buffer was added to each tube, and the cells were analyzed immediately by flow cytometry on a BD FACSVerse™. The results were analyzed with the software FlowJo 7.6.2. The membranes of early apoptotic cells are stained with Annexin V-FITC. Cells that are in late apoptosis or dead are stained with PI and Annexin V-FITC. The percentage of early apoptotic cells was calculated from three independent experiments.

### 2.11. BrdU Incorporation Assays

BrdU labelling was done using a BrdU Assay Kit (Roche, Basel, Switzerland). After treatment for 8 hours, the cells were incubated with 10 *μ*M BrdU labelling solution (diluted in culture medium) for 4 hours at 37°C. We removed the labelling solution and followed the protocol according to the instructions. We read the absorbance at 492 nm and 370 nm.

### 2.12. Data Analysis

Independent experiments were performed at different times. Similar results were obtained from at least three independent experiments for the statistical analysis. The data were analyzed using univariate ANOVA or Student's *t*-test. Statistical significance was set at *P* < 0.05.

## 3. Results

### 3.1. *C. albicans* Treatment Inhibits Autophagy Flux in THP-1-Derived Macrophages and RAW 264.7 Cells

**Figure 1:**
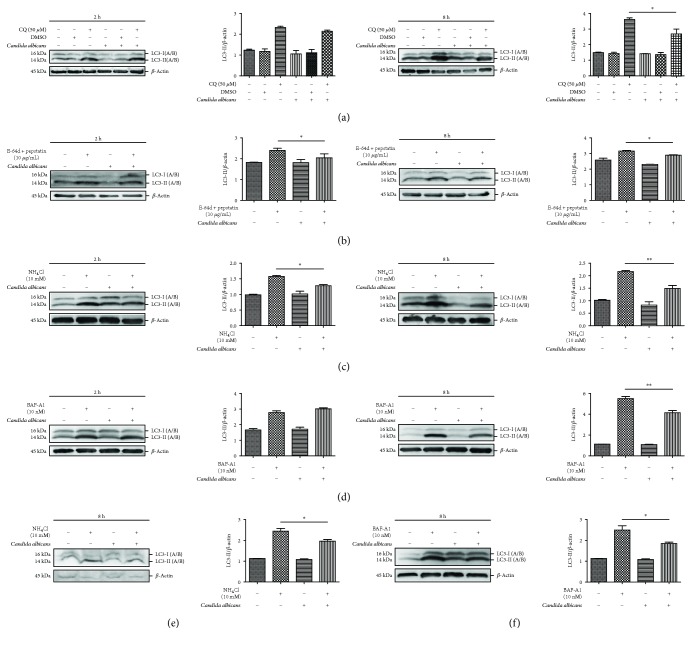
*Candida albicans* treatment reduces autophagic flux. (a) THP-1-derived macrophages were treated with *C. albicans* in the presence or absence of chloroquine (CQ) for the indicated time. The LC3-II/*β*-actin ratio decreased after treatment with *C. albicans*. A significant difference in the LC3-II/*β*-actin ratio was not observed between dimethyl sulfoxide- (DMSO-) treated and untreated cells. (b, c, d) THP-1-derived macrophages were treated with *C. albicans* in the presence or absence of E-64d+ pepstatin, NH_4_Cl, and BAF-A1 for the indicated period. The LC3-II/*β*-actin ratio decreased after treatment with *C. albicans*. (e, f) RAW 264.7 macrophage-like cells were treated with *C. albicans* in the presence or absence of NH_4_Cl/BAF-A1 for 8 hours. The LC3-II/*β*-actin ratio was decreased after treatment with *C. albicans.* Bars represent the mean ± SEM of *n* = 3 independent experiments performed in triplicate (^∗^*P* < 0.05; ^∗∗^*P* < 0.01).

The cytosolic form of LC3 (LC3-I) is converted to the phosphatidylethanolamine-conjugated form (LC3-II) during autophagy [25]. Thus, LC3 is generally used as the molecular marker to monitor autophagy level. The new LC3-II proteins assembled on the membranes of autophagosomes are degraded after the fusion of autophagosomes and lysosomes. To monitor autophagy flux, we measured LC3-II accumulation after treatment with *C. albicans* in the presence or absence of several lysosomal inhibitors, including E-64d + pepstatin, CQ, NH_4_Cl, and baflomycin-A1 (BAF-A1), that block autophagy flux at the final step. It is important to choose optimized treatment time conditions. Because the phagocytosis of *C. albicans* is expected to reach the peak at 1–3 h and complete at 6–8 h after stimulation with *C. albicans* in macrophages [26], so we set the detection time point as 2 h and 8 h after specific stimulation. We found that the level of LC3-II accumulation (LC3-II/loading control *β*-actin) was increased in THP-1-derived macrophages after treatment with lysosomal inhibitors CQ, E-64d + pepstatin, NH_4_Cl, or BAF-A1 for either 2 hours or 8 hours, indicating the basal autophagy level. Interestingly, the LC3-II accumulation was significantly decreased in THP-1-derived macrophages challenged with heat-killed *C. albicans* in the presence of CQ, E-64d + pepstatin, NH_4_Cl, or BAF-A1 for 8 hours compared to cells incubated with these lysosomal inhibitors alone. This finding suggested that *C. albicans* decreases LC3-II turnover in autophagy flux (Figures 1(a)–1(d)). Furthermore, we found that *C. albicans* stimulation decreased LC3-II accumulation in RAW 264.7 cells (mouse macrophage-like cell line) in the presence of NH_4_Cl or BAF-A1, compared to cells treated with NH_4_Cl or BAF-A1 alone (Figures 1(e)-1(f)). These data validated the results in the human cell line.

**Figure 2:**
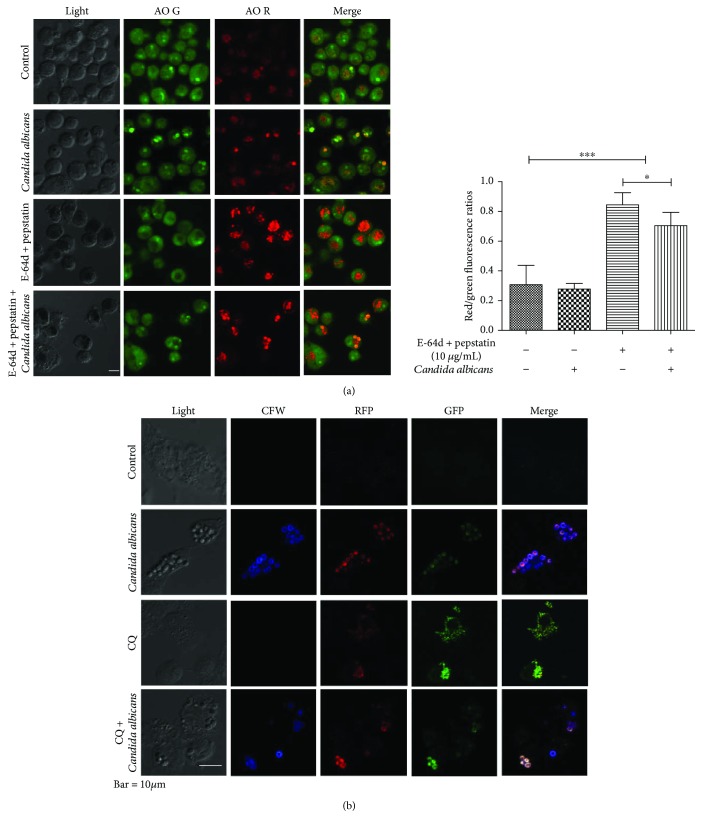
*Candida albicans* treatment reduces the autophagosome formation in THP-1-derived macrophages. (a) THP-1-derived macrophages were treated with *C. albicans* for 8 hours. Cells were then incubated with AO. (b) THP-1-derived macrophages were pretreated with RFP-GFP-LC3 before being treated with *C. albicans* (CFW stained) for 8 hours. The untreated THP-1-derived macrophages showed weak LC3 fluorescence. Chloroquine- (CQ-) treated THP-1-derived macrophages showed that LC3 gathered into clusters. The combined treatment of *C. albicans* and CQ weakened the clustering of LC3 (^∗^*P* < 0.05; ^∗∗∗^*P* < 0.001).

AO can stain acidic vacuoles, including lysosomes and autophagolysosomes. It is used in the autophagy assay as a supplementary method to monitor autophagy [27]. We found that treatment with E-64d and pepstatin for 8 hours increased the red/green fluorescence ratio in THP-1-derived macrophages. Intriguingly, the red/green fluorescence ratio declined in THP-1-derived macrophages challenged with *C. albicans* in the presence of E-64d and pepstatin compared to cells treated with E-64d and pepstatin alone. These data suggested that *C. albicans* decreased the formation of autophagolysosomes (Figure 2(a)).

The tandem RFP-GFP-LC3B assay was used to analyze autophagy flux and visualize cargo structures using fluorescence microscopy. GFP fluorescence is quenched by low pH, and RFP is more stable, so the combination of GFP and RFP fluorescence indicates the existence of autophagosomes that have not fused with lysosomes [28]. We found that CQ treatment for 8 hours increased the number of RFP puncta, GFP puncta and merged RFP-GFP puncta in THP-1-derived macrophages compared to control cells, suggesting that CQ blocked autophagy flux. We observed the significant phagocytosis of *C. albicans* in THP-1-derived macrophages by staining with calcofluor white (CFW). Importantly, RFP-GFP puncta were present around phagocytized *C. albicans* in THP-1-derived macrophages, and RFP-GFP puncta independent of *C. albicans* were rare. Although we still observed RFP-GFP puncta in THP-1-derived macrophages challenged with *C. albicans* in the presence of CQ, the RFP-GFP puncta independent of *C. albicans* were rare than those in cells treated with CQ alone. The above results indicated that LC3 protein was recruited to bind phagocytized *C. albicans* (Figure 2(b)).

### 3.2. *C. albicans* Treatment Induces LAP in THP-1-Derived Macrophages

**Figure 3:**
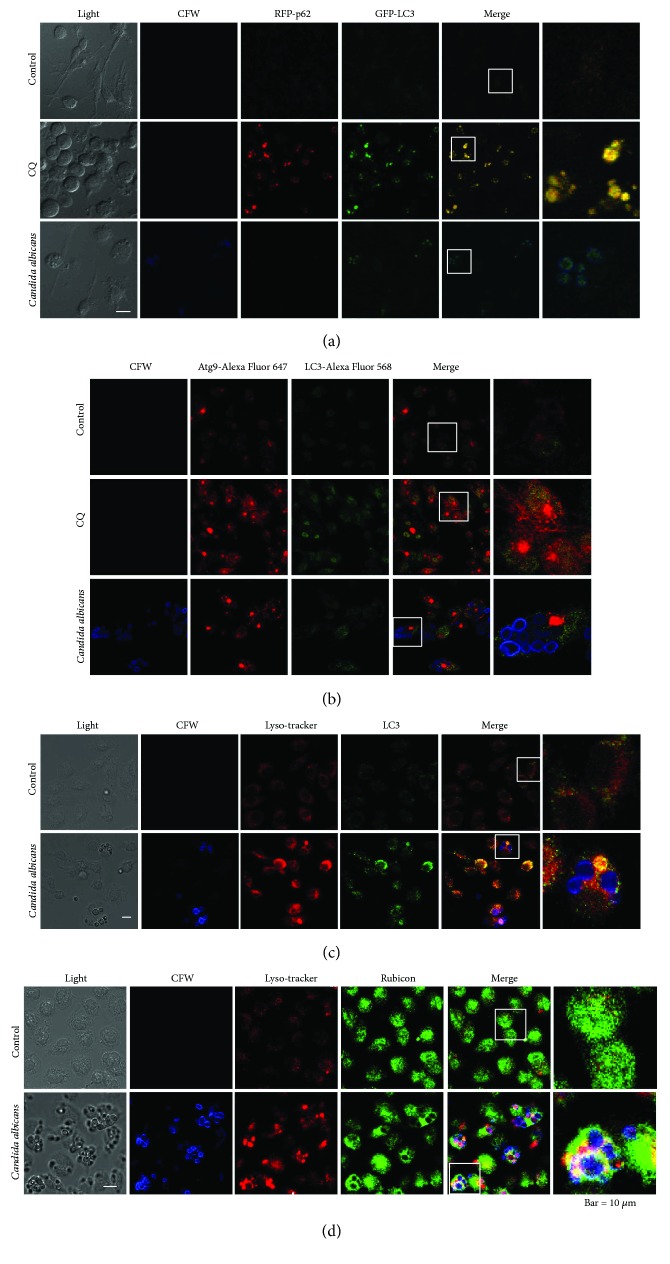
*Candida albicans* treatment induces LAP. (a) THP-1-derived macrophages were pretreated with GFP-LC3 and stimulated with *C. albicans* (CFW stained) for 8 hours. CQ-treated THP-1-derived macrophages showed LC3 and p62 colocalization and gathering into clusters. In *C. albicans*-treated cells, *C. albicans* spores colocalized with LC3 but without p62. (b) THP-1-derived macrophages were stimulated with *C. albicans* (CFW stained) for 8 hours. CQ-treated THP-1-derived macrophages harboured LC3 clusters, and the expression level of ATG9A increased. In *C. albicans*-treated cells, *C. albicans* spores colocalized with LC3 but without ATG9A. (c, d) BMDMs were treated with live *C. albicans* spores for 8 hours. The colocalization of Lyso-tracker and LC3 or Lyso-tracker and Rubicon was detected.

Our data indicated that *C. albicans* stimulation led to a decrease in LC3-II accumulation, while LC3 was significantly recruited around phagocytized *C. albicans*. In general, LC3 aggregation suggests autophagy. We speculated that the induction of LC3-associated phagocytosis contributed to the contradictory data. Therefore, we detected the colocalization of LC3 and autophagy-associated proteins to identify whether the recruitment of LC3 on *C. albicans* was involved in LAP or canonical autophagy regulation. The current knowledge is that LC3, beclin-1, PtdIns3KC3, and Atg12-Atg5-Atg16L are required for LAP, while p62, ATG9, and ULK1 are not needed for LAP [23, 29]. p62 is a linkage between LC3 and autophagic substrates that sends completed autophagosomes to fuse with lysosomes [30]. ATG9 is an essential ATG (autophagy-related gene) protein that participates in autophagosome formation and displays partial colocalization with LC3 [31]. We found that CQ treatment led to significant colocalization of GFP-LC3 and RFP-p62. In THP-1-derived macrophages challenged with *C. albicans*, we observed the recruitment of LC3 around phagocytized *C. albicans* without p62 or ATG9 involvement (Figures 3(a) and 3(b)).

We validated that *C. albicans* can induce LAP in wild-type bone marrow-derived macrophages (BMDMs) from C57BL/6 mice. In this assay, lysosomes were marked with a lysosomal tracker, and LC3 and Rubicon were visualized using immunofluorescence. We observed an increase in the colocalization of LC3 and lysosomes around phagocytized *C. albicans* in BMDMs (Figure 3(c)), suggesting LC3-associated transport to lysosomes. Moreover, Rubicon and lysosomes around phagocytized *C. albicans* were colocalized (Figure 3(d)). Given the critical role of Rubicon in the LAP process, our findings showed that the phagocytosis of *C. albicans* activated LAP in macrophages.

These data indicated that phagocytosis of *C. albicans* induced LAP, causing a significant recruitment of LC3 on phagocytized *C. albicans*, in a p62- and ATG9-independent manner, but this phagocytosis event inhibited the canonical autophagy process and decreased the aggregation of LC3 and p62.

### 3.3. *C. albicans* Challenge Inhibits Autophagy Flux via an MTOR-Independent Mechanism

**Figure 4:**
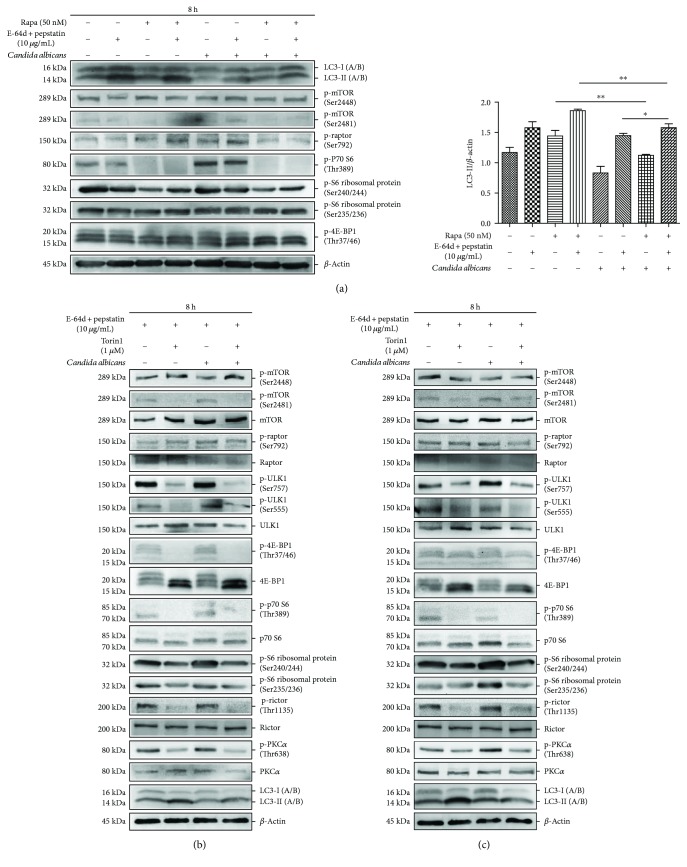
*Candida albicans* treatment decreases autophagy via an MTOR-independent mechanism. (a) THP-1-derived macrophages were treated with or without *C. albicans* and rapamycin for 8 hours in the presence or absence of E-64d and pepstatin. The level of LC3 protein was determined by Western blotting. The ratio of LC3-II/*β*-actin was calculated. (b, c) THP-1-derived macrophages were treated with or without *C. albicans* and torin1/pp242 for 8 hours. The level of MTORC1 and MTORC2 activity was detected using Western blotting. Bars represent the mean ± SEM of *n* = 3 independent experiments (^∗^*P* < 0.05; ^∗∗^*P* < 0.01).

Rapamycin is a widely used MTOR-dependent autophagy inducer. We found that rapamycin treatment decreased the phosphorylation levels of MTOR at Ser2481 and MTOR complex 1 substrates such as p70 S6 kinase at Thr389, S6 ribosomal protein at Ser 240/244, and 4E-BP1 at Thr37/46. These data suggested that the MTOR pathway is sensitive to rapamycin treatment (Figure 4(a)). Moreover, the LC3-II level was upregulated by rapamycin in the presence of E-64d and pepstatin compared to E-64d and pepstatin alone, indicating that rapamycin enhanced autophagy flux in THP-1-derived macrophages. Interestingly, *C. albicans* challenge did not increase the phosphorylation of MTOR or its substrates or reverse rapamycin-inhibited MTOR signalling. Importantly, LC3-II accumulation was decreased in cells challenged with *C. albicans* in the presence of rapamycin, E-64d, and pepstatin compared to those treated with rapamycin, E-64d, and pepstatin alone (Figure 4(a)), indicating that *C. albicans* challenge prohibited rapamycin-enhanced autophagy flux in an MTOR-independent manner. Therefore, our data demonstrated that the MTORC1 pathway did not participate in the *C. albicans*-induced decrease in autophagy flux. In addition, we found that the LC3-II level was increased in cells treated with *C. albicans* in the presence of rapamycin, E-64d, and pepstatin compared to those treated with *C. albicans* in the presence of E-64d and pepstatin alone, suggesting that rapamycin rescued slightly the *C. albicans*-inhibited autophagy flux.

Two MTOR inhibitors, torin1 and pp242, were used to confirm whether *C. albicans* stimulation affects the activity of both MTORC1 and MTORC2 [32, 33]. We found that torin1 and pp242 both inhibited the phosphorylation levels of MTORC1 (including ULK1 at Ser757, 4E-BP1 at Thr37/46, p70 S6 kinase at Thr389, and S6 ribosomal protein at Ser240/244) and MTORC2 (including Rictor at Thr1135 and protein kinase C at Thr638) and increased LC3-II accumulation. This finding indicated that both torin1 and pp242 can induce MTOR-dependent autophagy in THP-1-derived macrophages (Figures 4(b) and 4(c)). With these assays, we further validated that *C. albicans* did not increase the activity of MTORC1. In addition, *C. albicans* stimulation did not increase the phosphorylation of Rictor at Thr1135 or of protein kinase C at Thr638, nor did it prohibit the torin1- or pp242-induced inhibition on phosphorylation of MTOR complexes 1 or 2 (Figures 4(b) and 4(c)). Furthermore, LC3-II accumulation (in the presence of E-64d and pepstatin) was decreased in THP-1-derived macrophages stimulated with *C. albicans* in the presence of torin1 or pp242 compared to cells treated with torin1 or pp242 alone, suggesting that *C. albicans* decreased the torin1- or pp242-enhanced autophagy flux. Taken together, *C. albicans* challenge decreased the autophagy in THP-1-derived macrophages in an MTOR-independent manner.

### 3.4. *β*-Glucan Treatment Increases Autophagy Flux in THP-1-Derived Macrophages

Kyrmizi et al. [34] reported that *β*-glucan surface exposure of *Aspergillus fumigatus* conidia activates LAP. As the main component of the *C. albicans* cell wall, *β*-glucan is thought to be the major PAMP [35]. It serves as the initial stimulation when macrophages recognize and phagocytize *C. albicans*. Evidence indicates that *β*-glucan is involved in the *C. albicans*-induced LAP [9].

**Figure 5:**
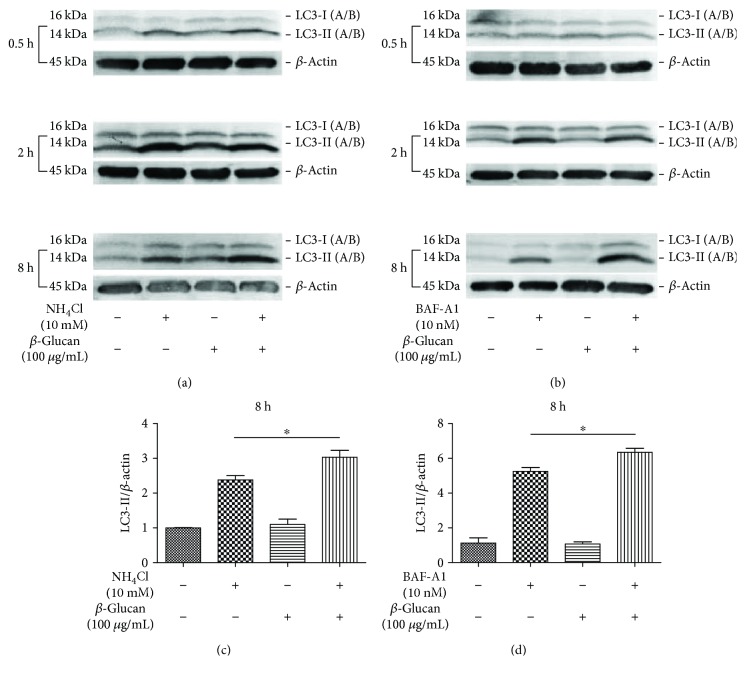
*β*-Glucan treatment increases autophagic flux in THP-1-derived macrophages. (a) THP-1-derived macrophages were treated with *β*-glucan in the presence or absence of NH_4_Cl for the indicated period. The LC3-II/*β*-actin ratio was increased after *β*-glucan treatment for 8 hours. (b) THP-1-derived macrophages were treated with *β*-glucan in the presence or absence of BAF-A1 for the indicated time. The LC3-II/*β*-actin ratio was increased after *β*-glucan treatment for 8 hours. Bars represent the mean ± SEM of *n* = 3 independent experiments (^∗^*P* < 0.05).

Interestingly, we found that *β*-glucan treatment increased the LC3-II level in the presence of BAF-A1 or NH_4_Cl compared with cells treated with BAF-A1 or NH_4_Cl alone (Figure 5). These data indicated that *β*-glucan increased LC3-II accumulation, and this finding is completely contrary to our results for cells treated with *C. albicans* in this study. The finding that *β*-glucan can increase LC3-II level is in accordance with a previous report [12]. We speculated that the phagocytosis and residence of *C. albicans*, but not *β*-glucan, triggered signalling regulation and led to the inhibition of autophagy flux.

### 3.5. LAP Induced by *C. albicans* Influences ROS Production, Apoptosis, and Proliferation of THP-1-Derived Macrophages

**Figure 6:**
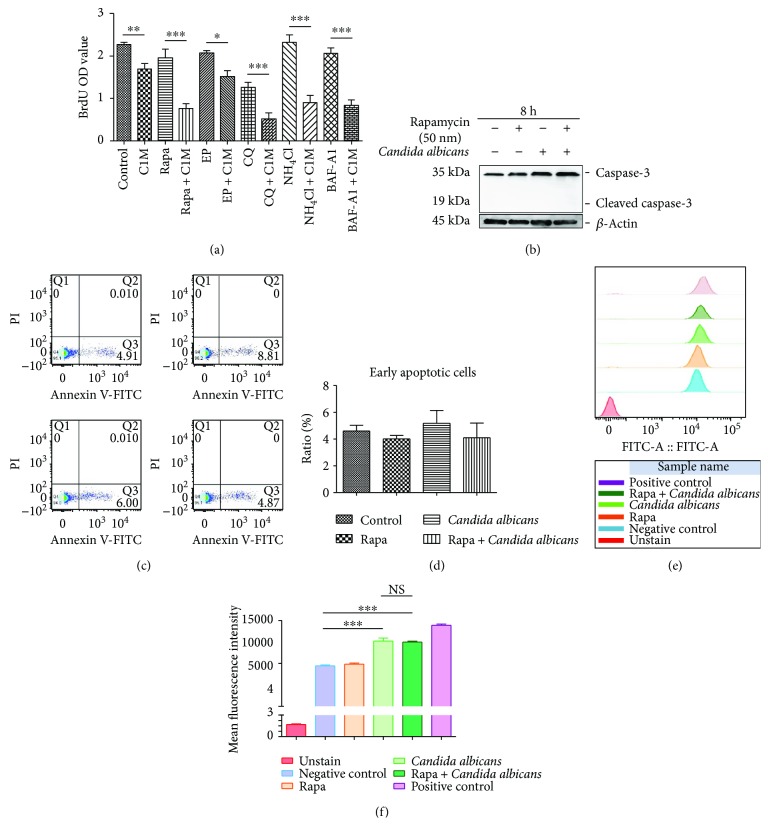
LAP induced by *Candida albicans* influences ROS production, apoptosis, and proliferation of THP-1-derived macrophages. (a) After treatment for 8 hours, proliferation of THP-1-derived macrophages was detected using the BrdU assay. The asterisks indicate significant differences. C1M, the *C. albicans* strain name. (b) THP-1-derived macrophages were treated with rapamycin in the presence or absence of *C. albicans* for 8 hours. Western blotting was used to determine the levels of caspase-3. (c, d) After treatment for 8 hours, cells were collected to detect apoptosis and death. The ratio of apoptotic cells was calculated, and there was no difference between groups. (e, f) After treatment for 8 hours, ROS production was assayed by flow cytometry. The mean fluorescence intensity (MFI) of DCFH-DA was analyzed, and the asterisks indicate significant differences. Bars represent the mean ± SEM of *n* = 3 independent experiments (^∗^*P* < 0.05, ^∗∗^*P* < 0.01, and ^∗∗∗^*P* < 0.001).

Autophagy is a key mechanism for maintaining intracellular homeostasis. Almost all mammalian cells have a basal level of autophagy activity. Therefore, we tested the biological impact of *C. albicans* stimulation. First, we found that *C. albicans* (C1M, the *C. albicans* strain name) challenge decreased BrdU incorporation (Figure 6(a)), indicating a decline in DNA synthesis. The autophagy flux blockers E-64d, pepstatin, CQ, NH_4_Cl, and BAF-A1 exhibited a distinct pattern of impact on DNA synthesis in THP-1-derived macrophages. E-64d, pepstatin, and BAF-A1 presented a slight inhibitory effect, and CQ presented a significant inhibitory effect. NH_4_Cl has a positive effect on DNA synthesis. Interestingly, when autophagy flux was blocked by lysosomal inhibitors, *C. albicans* stimulation led to a more significant inhibitory effect on DNA synthesis than when cells were challenged with *C. albicans* alone. These data demonstrated that nonobstructive basal autophagy might be essential for maintaining cell viability when macrophages have phagocytized *C. albicans*. Importantly, LAP is also involved in the degradation of lysosome-like canonical autophagy processes. Therefore, blocking lysosome activity is predicted to perturb LAP when autophagy flux is blocked. This conclusion needs to be validated by specific approaches to perturb LAP.

The balance between autophagy machinery and apoptosis regulation is essential for switching cell fate in response to various stresses. Thus, we detected apoptosis using flow cytometry with Annexin V and PI staining and Western blotting to identify caspase-3 cleavage. *C. albicans* stimulation, rapamycin treatment, and *C. albicans* stimulation in the presence of rapamycin did not cause cleavage of caspase-3 (Figure 6(b)) or an increase in Annexin V-positive cells compared to control (Figures 6(c) and 6(d)). These data indicated that phagocytosis of *C. albicans* inhibited autophagy flux and did not trigger apoptosis in THP-1-derived macrophages.

ROS generation is also an important event when cells suffer various injuries. We used DCFH-DA staining to show that *C. albicans* stimulation induced an increase in ROS in THP-1-derived macrophages. However, treatment with the autophagy inducer rapamycin did not affect ROS production in THP-1-derived macrophages challenged with *C. albicans* (Figures 6(e) and 6(f)).

Taken together, unconditioned operating basal autophagy flux is essential to maintain a low level of DNA synthesis in THP-1-derived macrophages challenged with *C. albicans*.

## 4. Discussion

Our study shows that phagocytized *C. albicans* leads to the inhibition of basal autophagic flux via MTOR-independent mechanisms in THP-1-derived macrophages. Moreover, autophagy plays an essential role in maintaining basal DNA synthesis in THP-1-derived macrophages challenged with *C. albicans*.

The autophagy pathway includes the formation of the phagophore, the initial sequestering compartment, the extension into an autophagosome, and the completion of the autophagosome, followed by fusion with lysosomes, degradation of the contents, and complete autophagic flux. The assay of LC3-II turnover using Western blotting in the presence or absence of lysosomal degradation is used to infer autophagic flux [36]. LC3-II will be more abundant in the presence of a lysosomal inhibitor when autophagic flux is occurring. Currently, the LC3-II turnover analysis mainly contributes to distinguishing actual autophagy induction from obstruction of autophagic flux [37]. This assay was clarified in the latest guidelines for the use and interpretation of assays for monitoring autophagy (published in December 2016). For example, higher LC3-II levels with some treatment plus lysosomal inhibitors compared to lysosomal inhibitors alone may indicate the enhancement of autophagic flux. If the treatment by itself upregulates LC3-II levels, but the treatment plus lysosomal inhibitors does not increase LC3-II levels compared to lysosomal inhibitors alone, then the treatment might cause a partial blockage in autophagic flux. Moreover, if a treatment by itself increases LC3-II levels without causing a difference in LC3-II levels when in combination with a lysosomal inhibitor compared to the treatment alone, this may indicate a complete blockage of autophagy at the final step. However, the guidelines did not discuss how to interpret the decline of autophagic flux. We treated THP-1-derived macrophages with *C. albicans* and lysosome inhibitors for 2 h and 8 h (the early and late time points of phagocytosis of *C. albicans*). Interestingly, LC3-II levels were increased in cells exposed to *C. albicans* and four lysosome inhibitors compared to *C. albicans* alone in our study. This finding indicated that the treatment did not block autophagic flux. Furthermore, lower LC3-II levels with *C. albicans* stimulation plus the lysosomal inhibitors compared to the lysosomal inhibitors alone suggest that *C. albicans* stimulation decreases autophagic flux. Our data of RFP-GFP-LC3B puncta analysis supported this conclusion. Autophagy inhibition was reported to contribute to the treatment of malignant diseases such as leukemia [38] and non-small-cell lung cancer [39]. Therefore, guidelines for the use and interpretation of assays for monitoring autophagy inhibition should be established, although the guidelines for monitoring autophagy induction have been updated consistently.

The fusion of the autophagosome and the lysosome is the crucial end of the autophagy process. Therapeutic approaches have targeted the lysosome and/or autophagosome and lysosome fusion to interrupt the autophagy process. E-64d and pepstatin are lysosomal inhibitors. CQ, NH_4_Cl, and BAF-A1 can neutralize lysosomal pH. In addition, BAF-A1 can block the fusion of autophagosomes and lysosomes. Importantly, these agents (CQ and BAF-A1 especially) have complex pharmacological effects (especially CQ) aside from regulating autophagy. We observed LC3-II accumulation in cells challenged with *C. albicans* in the presence or absence of all the above agents. As a result, we observed a decrease in LC3-II accumulation in the four groups. Our findings strongly argue that *C. albicans* led to a decrease in autophagic flux. Interestingly, our data demonstrate that different lysosomal inhibitors have different effects on blocking autophagic flux. In THP-1-derived macrophages, CQ and BAF-A1 had a stronger autophagy-blocking effect than E-64d plus pepstatin and NH_4_Cl. In contrast to E-64d plus pepstatin, CQ, NH_4_Cl, and BAF-A1 all exhibited a time-dependent increase in the inhibitory effect. However, in the four analysis systems in the presence or absence of lysosomal inhibitors, only E-64d plus pepstatin and NH_4_Cl promoted *C. albicans*-induced autophagy inhibition in the short treatment time of 2 hours. Although the treatment time of BAF-A1 or CQ was recommended to be short (1–4 hours), the two systems above could not show the effect of *C. albicans* stimulation for 2 hours. Our findings suggest that the increase or decrease in autophagic flux should be identified using at least three lysosomal inhibitors. Moreover, one should remember that lysosomal function is active in macrophages and that macrophage numbers are increased in several lysosomal storage diseases [40]. For monitoring autophagy in macrophages, the use of approaches for blocking lysosome should be emphasized for interpreting the results of treatment with multiple lysosomal inhibitors.

LAP is phagocytosis in a macrophage that is mediated by LC3 and single membrane-bound phagosomes containing pathogens. This process promotes the fusion of phagosomes with lysosomes. LAP can be observed by GFP-LC3 puncta and LC3 lipidation (increase in LC3-II) [37]. However, LAP and autophagy are difficult to distinguish due to the dependence of both processes on LC3. The available criteria for differentiating them include the following: (i) ROS production is involved in LC3 recruitment in LAP; (ii) LAP does not require ATG9, but autophagy (macroautophagy) of bacteria does require ATG9; and (iii) single-membrane structures are involved in LAP [37]. In our study, we observed that the ROS level was increased and that ATG9 and p62 were not involved in LC3 recruitment in THP-1-derived macrophages treated with *C. albicans*, suggesting the induction of LAP. In fact, *C. albicans* induction of LAP has been described in other studies [9, 12, 41]. In addition, the critical role of Rubicon in LAP was verified by other studies [22, 42]. In our study, we validated the colocalization of Rubicon and phagosome-engulfed *C. albicans*. Our findings showed that the detection of LC3 recruitment in situ (e.g., GFP-LC3 puncta around pathogens or LC3 detection by immunofluorescence) is a key method for monitoring LAP induction. It remains unclear whether LC3 turnover occurs in the LAP process. However, our study indicates that LC3 recruitment is a marker of LAP induction, but an increase in LC3-II levels is not. Therefore, the assays for LC3-I to LC3-II conversion and LC3-II turnover should be avoided when studying the LAP process. Moreover, LC3-II turnover was decreased after *C. albicans* stimulation-induced LAP in our study.

In conclusion, our study demonstrates that phagocytosis of *C. albicans* leads to a decrease in autophagy flux accompanied by the induction of LAP. The occupation of LC3 by recruitment to phagocytized *C. albicans* might contribute to the inhibition of basal autophagic flux. Our study reveals the coordinated machinery between canonical autophagy and LAP in defense against *C. albicans* challenge. Nonobstructive basal autophagy maintains cell viability when macrophages phagocytize *C. albicans*. The role of canonical autophagy in macrophages that participate in immune defense against *C. albicans* invasion must be clarified by specific approaches to depriving the autophagy machinery.

## Data Availability

The data used to support the findings of this study are available from the corresponding author upon request.

## Abbreviations

LAP: LC3-associated phagocytosis
PAMP: Pathogen-associated molecular pattern
PRRs: Pattern recognition receptors
LC3: Microtubule-associated protein 1 light chain 3
MTOR: Mechanistic target of rapamycin
MTORC1: MTOR complex 1
MTORC2: MTOR complex 2
AO: Acridine orange
CQ: Chloroquine
BAF-A1: Baflomycin-A1
ATG: Autophagy-related gene
PBS: Phosphate-buffered saline
CFW: Calcofluor white
BMDMs: Bone marrow-derived macrophages
ROS: Reactive oxygen species
ULK1: Unc-51-like kinase 1
DMSO: Dimethyl sulfoxide
FBS: Fetal bovine serum
PMA: Phorbol-12-myristate-13-acetate
WT: Wild-type
BSA: Bovine serum albumin
AVO: Acidic vesicular organelles
PI: Propidium iodide
EP: E-64d + pepstatin
Rapa: Rapamycin.
